# The intersection of sleep and synaptic translation in synaptic plasticity deficits in neurodevelopmental disorders

**DOI:** 10.1007/s00360-023-01531-3

**Published:** 2024-02-24

**Authors:** Rochelle L. Coulson, Philippe Mourrain, Gordon X. Wang

**Affiliations:** 1 Department of Psychiatry and Behavioral Sciences, Stanford University, Stanford, CA USA; 2 INSERM 1024, Ecole Normale Supérieure, Paris, France; 3 Wu Tsai Neuroscience Institute, Stanford University, Stanford, CA USA

**Keywords:** Sleep, Neurodevelopmental disorders, Synapse, Protein translation

## Abstract

Individuals with neurodevelopmental disorders experience persistent sleep deficits, and there is increasing evidence that sleep dysregulation is an underlying cause, rather than merely an effect, of the synaptic and behavioral defects observed in these disorders. At the molecular level, dysregulation of the synaptic proteome is a common feature of neurodevelopmental disorders, though the mechanism connecting these molecular and behavioral phenotypes is an ongoing area of investigation. A role for eIF2α in shifting the local proteome in response to changes in the conditions at the synapse has emerged. Here, we discuss recent progress in characterizing the intersection of local synaptic translation and sleep and propose a reciprocal mechanism of dysregulation in the development of synaptic plasticity defects in neurodevelopmental disorders.

## Introduction

Sleep is an evolutionarily conserved state. Sleep has essential roles in the health of an organism at the system level and sits at the intersection between many key molecular and metabolic pathways throughout the body and lifetime. Specifically, sleep has a critical developmental function in the nervous system on synaptic connections and plasticity. Sleep carries a major function in learning and memory consolidation (Benington and Frank 2003; Stickgold 2005; Diekelmann and Born 2010). Reactivation of neural circuits engaged during wake is part of the consolidation process during sleep, and this memory consolidation involves synaptic modification (Llinas and Steriade 2006; Born and Feld 2012). A net loss of synapses is found during sleep in the developing mouse cortex (Maret et al. 2011; Yang and Gan 2011), the zebrafish brain (Appelbaum et al. 2010), and the fly nervous system (Donlea et al. 2009; Bushey et al. 2011), indicating that sleep is also important for the downscaling of synaptic connectivity potentiated during wakefulness (Tononi and Cirelli 2003; Diering et al. 2017). Translation plays a critical role in synaptic plasticity that gives rise to memory consolidation, and proteins required for synaptic plasticity increase during the early hours of sleep (Aton et al. 2009). Sleep deprivation attenuates the initiation of mTORC1-dependent protein synthesis and impairs memory, which can be rescued by 4EBP2 phosphorylation (Tudor et al. 2016). The ability to regulate translation in cellular compartments distant from the nucleus, such as synapses, presents an additional challenge, which is facilitated by the ability to transport transcripts in anticipation of need for local translation at the synapse. This compartmentalization highlights the importance of translation as a spatio-temporal regulator of gene expression in the brain. Modulation from the nucleus alone is insufficient to respond to changes in distal synaptic environments. Sleep prioritizes the translation of proteins necessary for the repair of activity- or stress-induced damage that accumulates during wake (Cagnetta et al. 2019; Noya et al. 2019), thus persistent sleep deficits pose a significant threat to synaptic health and function.

Abnormal sleep is a common underlying feature of neurodevelopmental disorders (NDD). NDD is a broad classification of a wide variety of disorders which affect the proper development of the brain and other systems whose functions are intertwined with neuronal processes. Although these disorders stem from a broad spectrum of interacting genetic and environmental triggers, they converge on a set of core features, including cognitive impairment (Schwartz and Neri 2012), behavioral deficits (Bicks et al. 2015), abnormal sleep (Esbensen and Schwichtenberg 2016), and synaptic dysfunction (Ash et al. 2021; Golovin et al. 2021). Sleep abnormalities are prevalent amongst children with NDD, and 34% to 86% of children with intellectual disabilities experience sleep difficulties, which is thought to underlie the synaptic and behavioral deficits observed in these disorders (Limoges et al. 2005; Malow et al. 2006; Kronk et al. 2010; Sivertsen et al. 2012; Esbensen and Schwichtenberg 2016). Impaired sleep manifests as a variety of deleterious stresses and dysfunction at the molecular, cellular, and synaptic levels including altered DNA methylation and gene expression, redox metabolism, DNA damage repair, dendritic spine density, and synaptic plasticity (Vecsey et al. 2009; Narwade et al. 2017; Trivedi et al. 2017; Cedernaes et al. 2018; Ämmälä et al. 2019; Cheung et al. 2019; Raven et al. 2019; Coulson et al. 2022; Vanrobaeys et al. 2023). Although the genetic etiologies of NDDs are complex and diverse, mutations in two clusters of genes, those involved in translational regulation and in synaptic functions, are commonly observed in monogenic forms of autism spectrum disorder (ASD) (reviewed by Santini and Klann 2014). Concordance between the dysregulation in synaptic translation and sleep suggests sleep directly regulates synaptic plasticity and the development of behavioral and cognitive outcomes commonly observed in individuals with NDDs.

## Translational rhythms are sleep-dependent

The dichotomous regulation behind the seemingly synchronous patterns of transcription and translation in the mouse forebrain is divided between inherent and activity-driven rhythms. This relationship was demonstrated in a study by Noya et al (2019), which showed that synaptic transcripts and proteins both peak at two specific phases: pre-dawn and pre-dusk, however, the dependency of these oscillations on circadian rhythms and sleep are distinct (Noya et al. 2019). Transcripts involved in functions relating to translation and metabolism specifically peak prior to dawn, preceding the transition to the resting sleep phase in nocturnal mice. This contrasts with the pre-dusk peak, preceding the transition to the wake phase, which is enriched for synaptic signaling functions. Transcript oscillations persist under constant darkness conditions and are ablated in clock-deficient *Bmal1*^*−/−*^ mice; however, oscillations of many cycling transcripts are resistant to sleep deprivation, demonstrating strong circadian regulation at the level of transcription. Sleep supports macromolecule biosynthesis under conditions of stress in rodents, thus oscillations of the proteome are responsive to sleep state and conditions which promote sleep (Makletsova et al. 2006; Noya et al. 2019). 11.7% of synaptic proteins and 17.2% of forebrain proteins are rhythmic, and sleep deprivation ablates nearly all (98%) oscillating proteins, highlighting the contrasting sleep-dependent regulation of the synaptic proteome (Noya et al. 2019). Nearly half of locally translated synaptic proteins are represented in the oscillating proteome (Ouwenga et al. 2017). Among synaptically localized proteins, approximately 50% also exhibit cyclic phosphorylation, peaking at transitions between sleep and wake. Under conditions of sleep deprivation, 98% of rhythmic synaptic phosphorylation is lost (Brüning et al. 2019). The synchronous but differentially regulated relationship between the synaptic transcriptome and proteome suggests a model in which the production and transport of synaptic transcripts oscillate in circadian anticipation of need, followed by local translation at the synapse based on actual need during sleep and wake.

## Local translation enables spatio-temporal compartmentalization

**Fig. 1:**
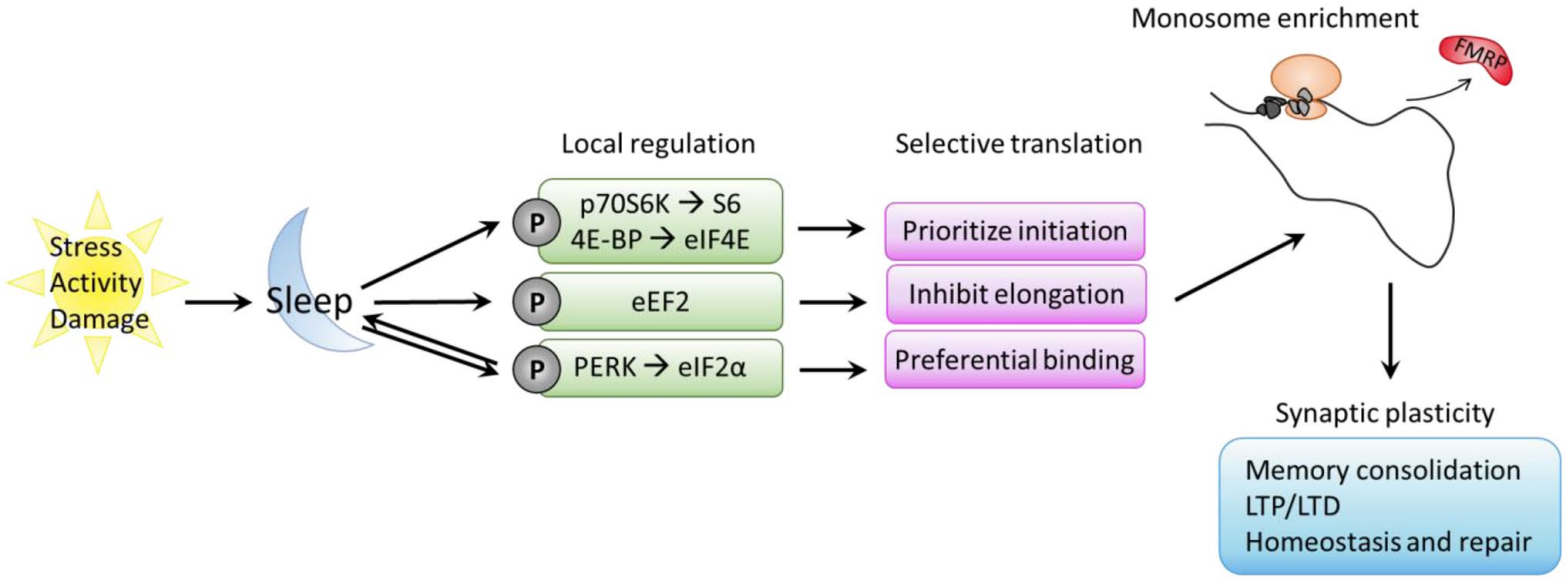
Translation is locally regulated during sleep to promote pathways involved in memory consolidation, homeostasis, and repair. Binding of specific translation regulators, prioritization of initiation over elongation, and preferential translation by monosomes promotes the synthesis of a diverse and specific pool of proteins required for these functions

Activity-dependent translation plays an important role in synapse maturation and function, which is critical in learning and memory (Migaud et al. 1998; El-Husseini et al. 2002). Increased need for translation efficiency in response to synaptic stimuli may require a shift towards local translation over protein shuttling. In recent years, evidence supporting local translation at synapses has grown, and it has become increasingly clear that protein synthesis occurs directly in pre- and postsynaptic compartments. Local translation responds to local activity and metabolic needs at large distances from the nucleus (Hafner et al. 2019). This offers increased flexibility in response to stimuli in distal cellular compartments, such as axons and dendrites. Transcripts and regulatory proteins are preemptively shuttled from the nucleus to sites of activity, with diversity in the 3’ untranslated region (UTR) playing a role in localization, stabilization, and translational regulation (Tushev et al. 2018). Locally translated transcripts are enriched for longer, more GC-rich coding sequences and UTRs with increased G-quartet structure (Ouwenga et al. 2017). This mechanism enables translation to occur directly on site by local pools of ribosomes and other translational machinery in response to stimuli and is critical for protein synthesis-dependent synaptic plasticity (Fig. 1).

The functional relationship between synaptic plasticity and translation has been demonstrated through reciprocal modulation of long-term depression (LTD) and long-term potentiation (LTP) by translation induction and inhibition respectively (Gkogkas et al. 2013; Santini et al. 2013). Furthermore, the importance of sleep-specific protein translation in memory consolidation and cortical plasticity has been demonstrated in ocular dominance plasticity (ODP) in the cat (Seibt et al. 2012). This model demonstrates that although transcription occurs during waking experience, protein translation must occur during sleep to promote ODP and favors the mTOR-dependent translation of a specific subset of plasticity-related transcripts. Sleep specifically promotes translation initiation over elongation through the phosphorylation of 4E-BP1 and eEF2, potentially enhancing the translation of specific pools of transcripts (Belelovsky et al. 2005; Seibt et al. 2012). To support proteomic needs at the synapse, polyribosomes transiently and selectively accumulate in dendritic spines during memory consolidation (Ostroff et al. 2017, 2018). However, within the neuropil, local translation prioritizes monosomes (solitary mRNA-associated ribosomes), compared to the soma, where translation by polysomes is more frequent. While the abundance of monosomes at the synapse was previously considered evidence for limited or inefficient local translation, this may be a mechanism of promoting the translation of a diverse set of proteins at the synapse, where ribosome availability may be limited. Neuropil transcripts translated by monosomes are enriched for functions relating to the synapse, vesicles, and dendritic tree and include both low and high abundance transcripts (Biever et al. 2020).

## Translation of stress response proteins is prioritized during sleep

RNA granules regulate the transport of aggregated mRNA–protein complexes for local translation at the synapse. Stress granules carrying mRNAs and binding proteins which function to reprogram translation to respond to stressful conditions may be particularly relevant to sleep-specific translation, as sleep promotes repair and recovery (Suberbielle et al. 2013; Bellesi et al. 2016; Xie et al. 2018; Cheung et al. 2019; Mourrain and Wang 2019; Zada et al. 2019). eIF2α is a central hub of translational regulation, mediating proteomic transitions between normal neuronal function and stress response. Phosphorylation of eIF2α results in a shift from polysome translation to monosomes (Bellato and Hajj 2016), thus after the buildup of damage from activity and stress during wake, phosphorylation of synaptically localized eIF2α may drive translation towards the prioritization of repair and recovery proteins by monosomes during sleep. Recently, a non-canonical translational program mediated by phospho-eIF2α was characterized in axons. This pathway is induced by Sema3A, which initiates an early wave of local translation by mTOR and ERK1/2, triggering the phosphorylation of eIF2α by PERK. This Sema3A-phospho-eIF2α pathway induces the translation of proteins involved in metabolic pathways, endoplasmic reticulum (ER) and mitochondrial processes, and response to stress (Cagnetta et al. 2019). This pathway, however, is distinct from the canonical stress response, resulting in an eIF2B-mediated upregulation of global translation rather than repression. In *Xenopus*, this local PERK-induced phospho-eIF2α translational pathway is required for axon guidance and terminal branching in the retina.

Not only does sleep dictate proteomic need, but proteostatic pathways also directly impact sleep–wake states. PERK signaling, which regulates translation in response to ER stress, promotes sleep in both *Drosophila* and zebrafish (Ly et al. 2020). PERK activity is directly linked to the phosphorylation of eIF2α, which is a critical component of translation initiation and is responsive to stress through the convergence of PERK, PKR, GCN1, and HRI pathways, known as the integrated stress response. Sleep deprivation induces PERK and eIF2α phosphorylation (Naidoo et al. 2005) and reduced ER stress improves sleep consolidation and cognitive performance (Hafycz et al. 2022). Additionally, Salubrinal, an inhibitor of eIF2α dephosphorylation, blocks LTP and promotes non-REM sleep (Costa-Mattioli et al. 2007; Methippara et al. 2009, 2012). Sleep is considered a restorative state, and changes in the translational profile is one mechanism by which this occurs. Oxidative stress, ER stress, and macromolecular damage all trigger responses in this translational pathway to restore proteostasis and prioritize the translation of proteins necessary to respond to a particular stress stimulus.

## Early life sleep has long-term behavioral effects

The developmental regulation of sleep is conserved across multiple species, including mammals, fish, birds, insects, and worms, with sleep duration peaking during early life and decreasing through development (reviewed by Kayser and Biron 2016). In humans, infants sleep 16–18 h a day on average, however, sleep architecture is markedly different during postnatal years compared to later in development, and is characterized by increased daytime sleep, fragmented nighttime sleep, and transition into REM sleep at onset, with 50% of sleep time spent in active sleep/REM sleep (Grigg-Damberger 2016). Sensory feedback during REM myoclonic twitches promotes cortico-hippocampal coherence and the development of sensorimotor circuits (Del Rio-Bermudez et al. 2020), and extended postnatal REM sleep likely plays a role in the early development of this system (Gómez et al. 2023). The human brain doubles in volume during the first year of life, a period of rapid synaptogenesis, reaching about 80–90% of adult volume by 2 years of age (Knickmeyer et al. 2008), and sleep duration during the first year of life is positively associated with this growth in brain volume (Pittner et al. 2023). This growth is mirrored by the increase of synaptic density and the abundance of synaptic proteins, which peak early in development (Glantz et al. 2007).

Early postnatal development is characterized by “critical periods”, or heightened periods of plasticity characterized by increased receptiveness to external stimuli. Sleep enhances plasticity during these periods (Frank et al. 2001; Wang et al. 2011) and synaptic changes caused by sleep disruption have long-term effects on behavior and cognition throughout life. Early postnatal sleep disruption leads to chronic age and sex-dependent dysregulation of sleep in adulthood and impaired sociability and social bonding in prairie voles (Jones et al. 2019; Jones-Tinsley et al. 2023). Similarly, early life sleep deprivation leads to long-lasting social novelty preference impairment in mice (Bian et al. 2022) and REM deprivation in neonatal rats leads to depressive symptoms in adulthood (Feng and Ma 2003). These deficits are characteristic of behavioral features shared by many NDDs, highlighting the impact of disrupted sleep during postnatal critical periods in the establishment of synaptic networks and shaping life-long changes in plasticity and behavioral outcomes.

## Sleep intervention may target synaptic translation defects in neurodevelopmental disorders

The pathology of many NDDs lies at the convergence of translational dysregulation, sleep abnormalities, and altered synaptic function. ASD is a complex NDD, characterized by social, behavioral, and cognitive deficits, and affects 1 in 59 children worldwide (Baio et al. 2018). The genetic etiology of ASD is extremely variable and often unknown, with combined copy number variant (CNV) and exome sequencing identifying single causative mutations in only 11% of simplex ASD cases (Sanders et al. 2015). Fragile X syndrome (FXS) is the most common monogenic cause of inherited intellectual disability and ASD, and is characterized by behavioral deficits, cognitive impairment, and sleep abnormalities (Kelleher and Bear 2008). FXS is primarily caused by the expansion of a CGG trinucleotide beyond 200 repeats within the 5ʹ UTR of the Fragile X Messenger Ribonucleoprotein 1 gene (*FMR1*), leading to its hypermethylation and silencing. Its encoded protein, Fragile X Messenger Ribonucleoprotein (FMRP), regulates approximately 4% of all mRNA transcripts in the brain (Brown et al. 2001), many of which play critical roles in synapse development and plasticity (Brown et al. 2001; Darnell et al. 2001; Miyashiro et al. 2003; Antar et al. 2006; Zalfa et al. 2007; Bassell and Warren 2008). Phelan-McDermid syndrome, another genetic cause of ASD, is caused by the loss of SHANK3, a junction protein critical for synaptic function, and shares many behavioral, cognitive, and sleep phenotypes with other NDDs (Peça et al. 2011; Ingiosi et al. 2019; Bian et al. 2022, 2023; Lord et al. 2022; Medina et al. 2022). Despite variability in the underlying etiologies of NDDs, poor sleep is an extremely pervasive feature, with negative consequences on brain development, cognition, mood, and behavior. Disrupted sleep is observed in infants prior to the development of autistic traits, suggesting a causal impact on the development of these traits (Reynolds et al. 2019; MacDuffie et al. 2020). Sleep onset in early development correlates with behavioral regulation in children with ASD (Tesfaye et al. 2021) and sleep difficulties are predictive of several diagnostic criteria including autism severity scores, social deficits, stereotypic behaviors, communication deficits, and general developmental abnormalities (Schreck et al. 2004). Although the causative mutation may vary, dysregulated protein synthesis is a commonly affected functional pathway among genetically unique cases (Table 1).

**Table 1:** Sl﻿eep and translation phenotypes are shared across many genetically distinct NDDs and likely contribute to synaptic and cognitive phenotypes

	Prevalence of sleep deficits	Sleep phenotypes	Translational phenotypes	Synaptic phenotypes	Cognitive and behavioral phenotypes
Autism spectrum disorder	86% (Liu et al. 2006)	Insomnia, bedtime resistance, parasomnias, sleep disordered breathing, morning rise problems, daytime sleepiness, increased sleep latency, decreased sleep efficiency, decreased REM, increased late-stage NREM (Liu et al. 2006)	Dysregulated translation (variable) (Lu and Hsueh 2021)	Abnormal mGluR5-mediated synaptic plasticity, increased dendritic spine density (variable, decreased in some models) (Nishiyama 2019)	Restrictive and repetitive behaviors, avoiding physical contact, communication deficits, sometimes non-verbal, social interaction deficits (Saxena and Chahrour 2017)

Fragile X syndrome	32–77% (Kronk et al. 2010; Richdale 2003)	Increased sleep latency, sleep fragmentation, reduced REM duration, fewer REM bouts, disrupted NREM (Kronk et al. 2010; Miano and Ferri 2010)	Enhanced or repressed translation depends on transcript features (Darnell et al. 2011; Greenblatt and Spradling 2018)	Overabundant dendritic spines, immature spines with long, thin morphology, excessive glutamate receptor internalization, enhanced mGluR-dependent LTD (Hodges et al. 2017)	Cognitive impairment, hyperactivity, anxiety, social avoidance, hyperarousal to stimuli, attention deficits, increased risk of ASD (Lozano et al. 2014)

Rett syndrome	80% (Boban et al. 2018)	Increased sleep latency, nighttime waking, fragmented sleep, impaired sleep rebound, sleep apnea, excessive daytime sleepiness, increased total sleep time, abnormal REM/NREM rhythm (Boban et al. 2018)	Reduced global translation (Rodrigues et al. 2020)	Decreased dendritic spine density, impaired dendritic arborization (Lo and Lai 2020)	Regression of learned abilities, epileptic seizures, impaired nociception, stereotypic hand movements, poor response to environmental stimulation, impaired cognitive, social, and motor skills (Nomura 2005)

Down syndrome	65% (90% sleep apnea) (Horne et al. 2019)	Sleep apnea, increased sleep latency, frequent night awakening, parasomnias, fragmented sleep, reduced REM, daytime sleepiness, decreased sleep efficiency, decreased NREM stage 2 (Horne et al. 2019; Heubi et al. 2021)	Disrupted proteostasis through activation of the integrated stress response (ISR) translational pathway (Zhu et al. 2019)	Reduced spine density, larger dendritic spine heads, reduced cortical dendritic branching, spine maturation deficits, impaired synaptogenesis (Lauterborn et al. 2020)	Impaired memory, hyperactivity, intellectual disability, increased risk of Alzheimer's disease, delayed expressive language and verbal deficits, decreased anxiety, impaired attention, perception, and motor skills, sensory impairment, seizures (Grieco et al. 2015)

Local translation in dendritic spines plays an important role in their size and morphology, which is a major molecular phenotype of ASD. Overexpression of eIF4E, a key factor in translation initiation, alone mimics many synaptic and behavioral phenotypes of ASD in mice and is rescued by the downregulation of translation or specific knockdown of neuroligins (Gkogkas et al. 2013). In fact, disruption of the synthesis of several synaptic proteins, resulting in either overexpression or under expression, leads to the development of ASD-like phenotypes (Santini et al. 2013). In dendrites and dendritic spines, FMRP is involved in mRNA transport and the local synthesis of proteins involved in postsynaptic functions (Feng et al. 1997b, a; Weiler et al. 2004; Dictenberg et al. 2008). FMRP functions as part of ribonuclear protein (RNP) granules to regulate translation in cellular compartments distant from the nucleus, such as synapses. FMRP preferentially binds and promotes the translation of large transcripts, which often have low translation efficiencies, similar to transcripts that are preferentially translated by phospho-eIF2α and transcripts that are locally translated. Concordantly, locally translated transcripts are enriched for FMRP binding (Ouwenga et al. 2017). In *Drosophila*, dFmr1 plays an important role in the translation of transcripts supporting neurogenesis after a prolonged pause at the oocyte stage (Greenblatt and Spradling 2018). Thus, FMRP preserves the translational efficiency of targets that must undergo delayed translation after transport or storage. This stimulus-induced delayed translation is essential for modulation of neuronal networks, such as the plasticity of synaptic strength through the regulation of glutamate signaling at the synapse. FMRP modulates the translation of specific mRNA pools that directly affect the internalization of glutamate receptors at the synapse (Bear et al. 2004; Bhakar et al. 2012). Glutamate receptor internalization is a critical step in LTD-dependent synaptic plasticity (Bear et al. 2004) and in normal sleep-dependent synaptic homeostasis (Bushey et al. 2011).

In *Drosophila*, dFmr1 levels are inversely correlated with sleep amount, with high levels corresponding to short sleep and low levels corresponding to long sleep, and modulation in both directions impairs sleep homeostasis after deprivation (Bushey et al. 2009). Disrupted rhythms of metabolic demand at the synapse due to persistent sleep dysregulation likely has significant impacts on synaptic function and behavioral and cognitive outcomes. Targeting of major translational regulators presents a potential therapeutic strategy for the rescue of synaptic and behavioral phenotypes observed broadly among NDDs. Because of the shift in proteomic need between wake and rest, sleep is one of the key drivers of translation. Failure to coordinate proteomic levels with energy demand hinders the ability of synapses to cycle between activity and repair. Thus, the frequent and persistent sleep deficits experienced by individuals with NDDs likely cause a detrimental shift in translation, leading to an imbalance in the synaptic proteome and the development of adverse behavioral and cognitive outcomes. Sleep defects in NDDs manifest very early in development, likely having a compounding effect on synaptic structure and connectivity over the course of an individual’s life. Combining pharmacological treatment with sleep intervention early in development is a promising therapeutic strategy in modulating local synaptic translation and improving cognitive and behavioral outcomes in NDDs.

## Discussion

**Fig. 2:**
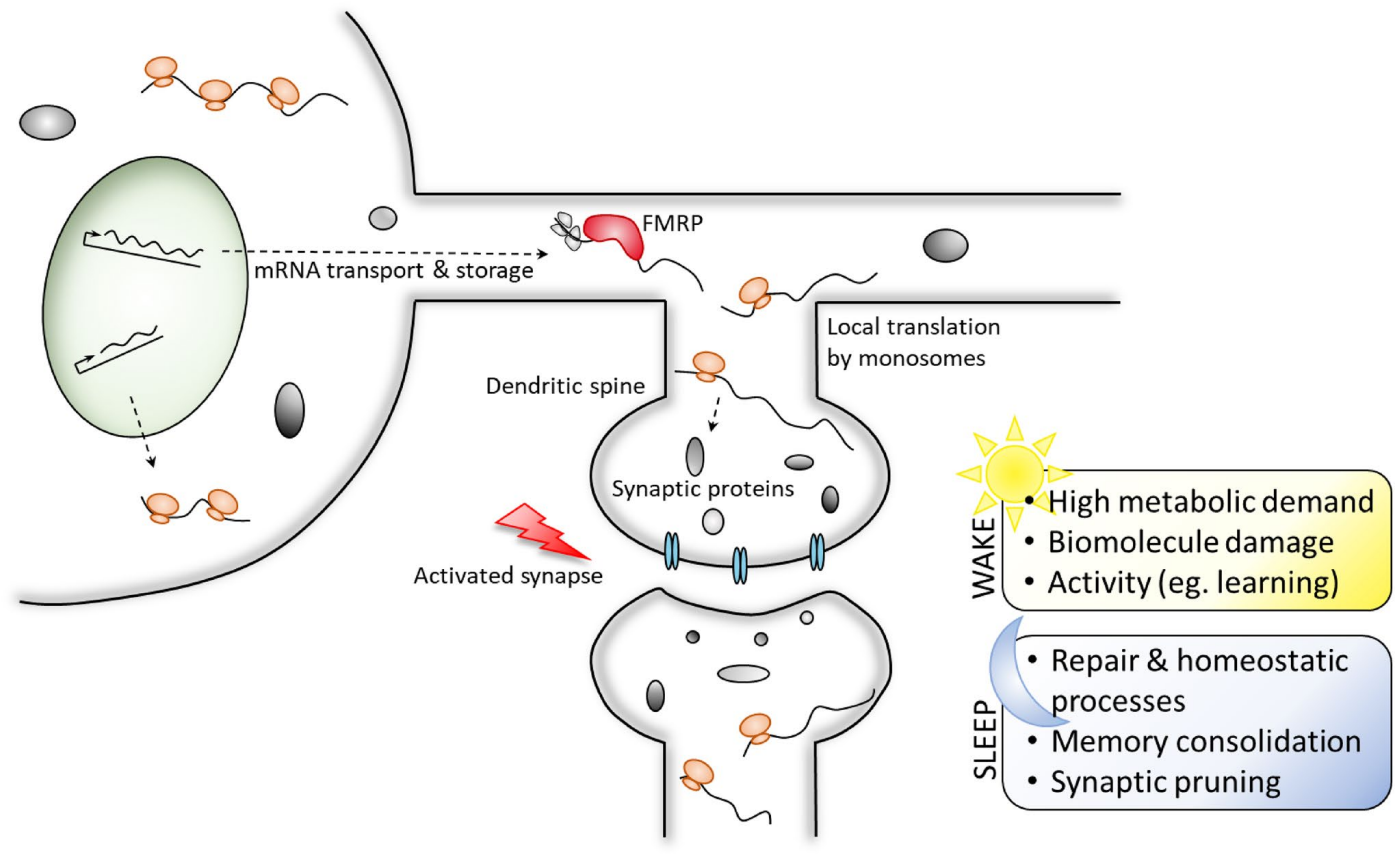
Local translation at the synapse responds to changes in proteomic need between wake and rest, enabling synapses to shift between states of metabolically demanding activity and repair and maintenance

Sleep is a fundamental state, conserved across all animals and plays essential roles in functions throughout the body, including cellular metabolism, biomolecular repair, and synaptic plasticity. In particular, the awake brain is highly metabolically demanding and is under unique regeneration constraints. Sleep provides a restorative state to maintain and repair this system, however, the specific mechanisms driving sleep-mediated synaptic plasticity and how they are dysregulated in disease are not fully understood. Further study examining the molecular mechanisms of sleep-dependent synaptic translation is critical for the development of targeted therapies to improve the quality of life of individuals with NDDs. Sleep intervention presents a non-invasive and adaptable therapeutic strategy to alleviate the cognitive and behavioral phenotypes that arise from dysregulation at the molecular level. Local translation at the synapse provides the spatial and temporal capacity necessary to respond to changes in signaling and metabolic demand between states of activity and rest (Fig. 2). Additional research on the role of eIF2α-mediated translation at the synapse in the response to acute stress and physiological rhythms of activity/rest and damage/repair will provide valuable insight into the role of this translational hub in supporting synaptic plasticity and cognitive function. The complexity of the synaptic network creates increased demand on translational regulation, and sleep provides a critical phase for the processing of waking experiences and repair of activity-induced damage, which rely heavily on protein synthesis. Impaired sleep results in a variety of stresses and dysfunction at the molecular, cellular, and synaptic levels. While sleep deficits are widely thought to contribute to cognition and memory impairment in NDD, the molecular underpinnings of its effect on synaptic plasticity are complex and not fully understood. Although NDDs arise through diverse genetic and environmental interactions and differ in their unique presentation, disruption of translation by sleep dysregulation could be a core phenotype across the spectrum.
